# Синдром переустановки осмостата — когда гипонатриемия становится «нормой»: аспекты диагностики, описание клинического случая

**DOI:** 10.14341/probl13235

**Published:** 2023-11-11

**Authors:** Л. И. Астафьева, И. Н. Бадмаева, И. С. Клочкова, Ю. Г. Сиднева, О. И. Шарипов, О. А. Гаджиева, Б. А. Баширян, П. Л. Калинин, А. Ю. Лубнин, А. Н. Коновалов

**Affiliations:** Национальный медицинский исследовательский центр нейрохирургии имени академика Н.Н. Бурденко; Национальный медицинский исследовательский центр нейрохирургии имени академика Н.Н. Бурденко; Национальный медицинский исследовательский центр нейрохирургии имени академика Н.Н. Бурденко; Национальный медицинский исследовательский центр нейрохирургии имени академика Н.Н. Бурденко; Научно-исследовательский институт неотложной детской хирургии и травматологии; Национальный медицинский исследовательский центр нейрохирургии имени академика Н.Н. Бурденко; Национальный медицинский исследовательский центр нейрохирургии имени академика Н.Н. Бурденко; Национальный медицинский исследовательский центр нейрохирургии имени академика Н.Н. Бурденко; Национальный медицинский исследовательский центр нейрохирургии имени академика Н.Н. Бурденко; Национальный медицинский исследовательский центр нейрохирургии имени академика Н.Н. Бурденко; Национальный медицинский исследовательский центр нейрохирургии имени академика Н.Н. Бурденко

**Keywords:** сброс осмостата, гипонатриемия, гипернатриемия, осмоляльность, натрий, синдром неадекватной секреции антидиуретического гормона

## Abstract

Синдром переустановки осмостата (СПО) характеризуется изменением нормального порога осмоляльности плазмы (уменьшением или повышением), что приводит к развитию хронической диснатриемии (гипо- или гипернатриемии). Мы описали клинический случай развития СПО, сопровождающегося хронической гипонатриемией, у пациентки с хордоидной глиомой III желудочка. Известно, что у пациентки ранее выявлялась гипонатриемия (131–134 ммоль/л). В ходе обследования были исключены гипотиреоз и гипокортицизм. Подтверждена сохранная фильтрационная функция почек (расчетная скорость клубочковой фильтрации CKD-EPI 91,7 мл/мин/1,73 м2). Для исключения нарушения концентрационной функции почек исследованы осмоляльность и уровень натрия мочи. Также были использованы расчетные формулы натрийуреза, которые показали отсутствие повышенной почечной экскреции натрия. В первые 3 сут после удаления опухоли третьего желудочка (хордоидная глиома, WHO Grade II) уровень натрия снизился до 119 ммоль/л; неоднократные инфузии гипертонического 3% раствора натрия хлорида в объеме 200–300 мл, глюко- и минералокортикоидная терапия не дали значимого эффекта, повышая показатели натрия плазмы на 2–3 ммоль/л с возвращением к исходному уровню в течение 6–8 ч. Гипопитуитарные нарушения после операции не развились. При дальнейшем наблюдении в течение 6 мес после операции уровень натрия сохранялся в пределах 126–129 ммоль/л. Проба с водной нагрузкой позволила исключить классический синдром неадекватной секреции антидиуретического гормона и подтвердила диагноз СПО. Учитывая отсутствие клинических симптомов, связанных с гипонатриемией, медикаментозной коррекции не потребовалось, пациентке рекомендовано дальнейшее амбулаторное наблюдение.

## Abstract (en)

Reset osmostat syndrome (ROS) is characterized by a change of normal plasma osmolality threshold (decrease or increase), which leads to chronic dysnatremia (hypo- or hypernatremia). We have described a clinical case of ROS and chronic hyponatremia in a patient with chordoid glioma of the III ventricle. It is known that the patient had previously been diagnosed with hyponatremia (131–134 mmol/l). She has not hypothyroidism and hypocorticism. There is normal filtration function of the kidneys was (CKD-EPI 91.7 ml/mi/1,73m2). Urine osmolality and sodium level were studied to exclude of concentration kidney function disorder. During first three days after removal of the tumor of the third ventricle (chordoid glioma, WHO Grade II), the sodium level decreased to 119 mmol/l. Repeated infusions of 200–300 ml hypertonic 3% sodium chloride solution, gluco- and mineralocorticoid therapy was ineffective, increasing plasma sodium levels by 2–3 mmol/l with the return to the initial level during 6–8 hours. Hypopituitary disorders did not develop after surgery. With further observation, the sodium level remained within 126–129 mmol/l for 6 months after surgery. The water load test make exclude the classic syndrome of inappropriate secretion of antidiuretic hormone, and confirmed the diagnosis of RSO. Because of absence of clinical symptoms associated with hyponatremia, no medical correction was required, patient was recommended to clinical follow-up.

## ВВЕДЕНИЕ

Гипонатриемия — распространенное электролитное нарушение у госпитализированных пациентов, наиболее часто встречающееся при нейрохирургических заболеваниях. При лечении гипонатриемии необходимо правильно установить ее генез, что зачастую бывает непросто. Как известно, одной из причин гипонатриемии является синдром нарушения секреции антидиуретического гормона, который, как правило, самостоятельно разрешается, реже переходит в хроническую форму. В литературе описаны несколько типов нарушения секреции антидиуретического гормона (АДГ) при данной патологии. Одним из наиболее редко встречающихся нарушений является cиндром переустановки осмостата (СПО), при котором изменяется нормальный порог осмоляльности плазмы, что становится причиной хронической гипонатриемии.

В представленной статье мы проводим собственное клиническое наблюдение данной патологии.

## ОПИСАНИЕ КЛИНИЧЕСКОГО СЛУЧАЯ

Пациентка З., 44 года, обратилась в НМИЦ нейрохирургии им. Н.Н. Бурденко в апреле 2022 г. с результатами проведенных исследований для определения тактики лечения.

Из анамнеза известно, что в марте 2022 г. пациентка была госпитализирована в стационар с токсической эритемой, разрешившейся на фоне антигистаминной терапии. Хронические заболевания пациентка отрицает. При обследовании обращала на себя внимание выраженная гипонатриемия, уровень натрия составлял 118 ммоль/л (референсные значения — 135–145). На фоне проведения инфузионной терапии 3% раствором натрия хлорида, приема петлевых диуретиков (фуросемид) и глюкокортикоидов (гидрокортизон) отмечался временный эффект с повышением уровня натрия плазмы до 125–130 ммоль/л. Учитывая неясный генез гипонатриемии, была проведена спиральная компьютерная томография головного мозга, в результате которой было выявлено супраселлярное новообразование в проекции третьего желудочка. При детальном расспросе выяснено, что в феврале 2022 г. пациентка отмечала эпизоды жажды до 4 л/сут и частого мочеиспускания, которые самостоятельно регрессировали в течение месяца. Других диэнцефальных проявлений не наблюдалось. Для уточнения диагноза и выбора тактики лечения пациентка была направлена в НМИЦ нейрохирургии им. Н.Н. Бурденко.

При поступлении состояние удовлетворительное. Рост 169 см, масса тела 62 кг, индекс массы тела (ИМТ) — 21,7 кг/м² (норма 18–25 кг/м²). Кожные покровы и видимые слизистые нормальной влажности и окраски. Подкожно-жировая клетчатка развита умеренно, распределение равномерное. АД 110/70 мм рт.ст. ЧСС 68 в минуту. Периферических отеков нет. Дизурических явлений не отмечено, суточный диурез составлял около 2000 мл/сут. Жажды не было.

Постоянно лекарственной терапии не получала.

Гинекологический анамнез: в течение жизни менструальный цикл регулярный. Беременностей не было, пациентка использовала контрацепцию.

При нейроофтальмологическом обследовании признаков воздействия на зрительный путь на основании головного мозга не выявлено. При осмотре психиатром отмечена эмоционально-личностная недостаточность, проявляющаяся малой инициативностью, апатичностью, недостаточностью осознания своего состояния. Неврологического дефицита при осмотре неврологом не было выявлено.

В гормональном анализе крови при поступлении уровень кортизола крови утром 405 нмоль/л (101–536), что позволило исключить надпочечниковую недостаточность. Других гипопитуитарных нарушений также выявлено не было: пролактин 109 мкМЕ/мл (110–562), ТТГ 1,58 мМЕ/мл (0,4–4), Т4 свободный 13,3 мМЕ/мл (9–19).

В биохимическом анализе крови отмечена умеренная гипонатриемия — 131–135 ммоль/л (135–145). Фильтрационная функция почек не нарушена, креатинин — 69 мкмоль/л (50–98), расчетная скорость клубочковой фильтрации CKD-EPI — 91,7 мл/мин/1,73 м². Исследование на осмоляльность крови и мочи до операции не выполнялось. В клиническом анализе мочи удельная плотность составила 1012 г/л. Углеводный обмен не нарушен, уровень гликированного гемоглобина — 4,8% (<6,0%).

Патологических отклонений при ЭКГ не зафиксировано, ритм синусовый, ЧСС 70 в минуту.

По данным магнитно-резонансной томографии головного мозга с внутривенным контрастированием в проекции III желудочка определялось крупных размеров образование с неоднородной структурой с наличием кист и перифокального отека, вызывающее компрессию и смещение хиазмы. После введения контраста отмечалось выраженное накопление контрастного вещества самим образованием и слабо выраженное в стенке кисты (рис. 1).

**Figure:**
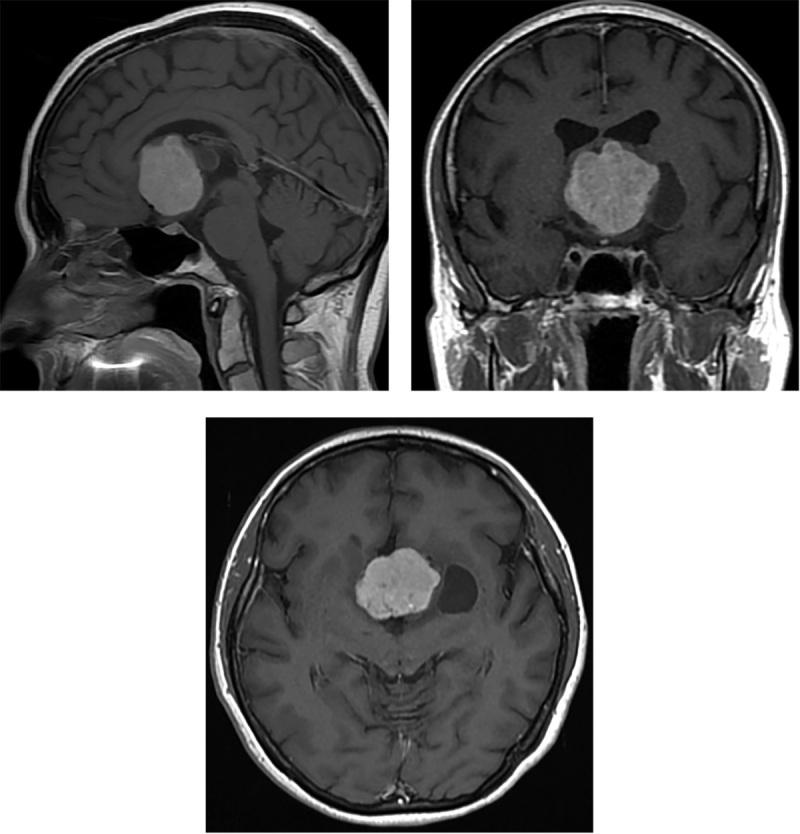
Рисунок 1. МРТ головного мозга с контрастированием пациентки З. Визуализируется крупных размеров образование в проекции третьего желудочка с неоднородной структурой, наличием кист и перифокального отека, вызывающее компрессию и смещение хиазмы.

21.06.2022 проведена операция (хирург — акад. РАН А.Н. Коновалов), удаление опухоли третьего желудочка транскаллезным доступом с нейрофизиологическим мониторингом. Осуществлен доступ к правому боковому желудочку через мозолистое тело. В ходе операции обнаружена плотная опухоль, местами хрящевой плотности, выполняющая отверстие Монро с распространением в крышу и базальные отделы III желудочка. Произведено лишь частичное удаление опухоли III желудочка, обусловленное исключительной плотностью, удаленными остались лишь верхние и задние фрагменты опухоли, больше с левой стороны. Результат срочной биопсии показал доброкачественную глиальную опухоль. В связи с характером опухоли и инфильтрацией прилежащей ткани попытка удаления ее целиком не предпринималась.

Гистологическое исследование подтвердило хордоидную глиому, WHO Grade II.

В раннем послеоперационном периоде проводилась терапия дексаметазоном 8 мг/сут в/м в течение 3 сут, далее гидрокортизоном перорально (20 мг/сут), препаратами гормонов щитовидной железы (L-тироксин 50 мкг перорально). В 1-е сутки после операции вечером отмечена тенденция к снижению уровня натрия до 129 ммоль/л, к терапии был добавлен препарат флудрокортизона (Кортинефф) 100 мкг перорально 2 раза в день.

Несмотря на проведение глюко- и минералокортикоидной терапии, к 3-м суткам после операции уровень натрия снизился до 119 ммоль/л, что совпало с усилением выраженности эмоционально-личностных и когнитивных расстройств (некритичность, снижение памяти на текущие события).

Инфузии 3% раствором натрия хлорида в объеме 200–300 мл на фоне терапии глюко- и минералокортикоидами приносили кратковременный эффект, повышая показатели уровня натрия плазмы на 2–3 ммоль/л с возвращением к исходному в течение 6–8 ч. Рекомендации по ограничению жидкости выполнялись пациенткой частично ввиду снижения критики к своему состоянию, суточный диурез оставался в пределах 2000–2500 мл, объем выпитой жидкости соответствовал выделенной.

На 10-е сутки после операции отмечено появление отечности нижних конечностей, которая регрессировала на фоне терапии петлевыми диуретиками. Постепенно уменьшилась степень выраженности эмоционально-личностных и когнитивных нарушений, которые выявлялись в той же степени, что и до операции: сохранялись снижение запоминания, забывчивость, малая инициативность, снижение активности, недостаточность осознания своего состояния. При выписке после отмены гормональных препаратов при проведении гормонального анализа крови данных за надпочечниковую недостаточность не получено: кортизол крови утром 431 ммоль/л (101–536). На момент выписки отмечалась кратковременная нормализация уровня натрия 135 ммоль/л (135–145), с последующим снижением до 130 ммоль/л через 3 дня после выписки.

Через 1 мес после операции выполнен биохимический анализ крови и мочи (табл. 1), гормональный анализ крови: пролактин 163 мкМЕ/мл (110–562), ТТГ 0,77 мЕд/л (0,40–4,00), T4 свободный 11,5 пмоль/л (9,0–19,0), кортизол 164 нмоль/л (101–536), эстрадиол 129,0 пмоль/л. Была проведена проба с водной нагрузкой. После приема 1000 мл (из расчета 15 мл на 1 кг веса) чистой столовой воды в течение 4 ч пациентка выделила 690 мл мочи (69%), в течение 5 ч — 840 мл (84%). Исследование осмоляльности и натрия не проводилось.

**Table:** Таблица 1. Биохимический анализ крови и мочи пациентки З.

Биохимический анализ крови
Показатель	Результат	Референсный интервал
Ферритин, мкг/дл	24,5	4,6–204
Натрий, ммоль/л	128	136–145
Осмоляльность, мОсмоль/кг	262	280–300
Калий, ммоль/л	4,7	3,5–5,1
Хлор, ммоль/л	93	98–107
Глюкоза, ммоль/л	5,4	3,9–5,8
Креатинин, мкмоль/л	64	50–98
Мочевина, ммоль/л	3,9	2,5–6,7
Мочевая кислота, мкмоль/л	126	210–420
Общий белок, г/л	74	64–83
Альбумин, г/л	44	35–52
АЛТ, Ед/л	21	0–55
АСТ, Ед/л	19	5–34
Кальций общий, ммоль/л	2,15	2,1–2,55
Кальций ионизированный, ммоль/л	1,15	1,09–10,3
Фосфор, ммоль/л	1,27	0,74–1,52
Железо, мкмоль/л	13,3	9–30,4
С-реактивный белок, мг/л	0,9	0–5
Биохимический анализ разовой порции мочи
Натрий, ммоль/л	91	40–220
Калий, ммоль/л	21,3	25–125
Хлор, ммоль/л	77	110–250
Кальций, ммоль/л	2,84	2,5–7,5
Мочевина, ммоль/л	154	428–714
Мочевая кислота, ммоль/л	0,643	1,48–4,43
Глюкоза, ммоль/л	0,1	0,1–0,8
Креатинин, ммоль/л	3,0	4,2–9,7
Белок, ммоль/л	0,02	0,01–0,14
Осмоляльность, мОсм/кг	399	300–1200
Фракционная экскреция мочевой кислоты (FEUA), %	10,2	4–11

В течение 3 последующих месяцев сохранялась гипонатриемия в пределах 128–131 ммоль/л на фоне удовлетворительного клинического статуса, соответствующего дооперационному. Гипопитуитарных нарушений не наблюдалось, уровень кортизола крови 358–401 нмоль/л (101–536).

После операции проведен курс стереотаксической конформной лучевой терапии на остаточную опухоль III желудочка с краевым захватом 5 мм (объемом 111,7 см³), подведено 30 фракций с РОД=1,8 Гр до средней СОД=54 Гр на ЛУЭ «ТруБим» с использованием технологий VMAT — методикой динамических арок (с 3 арок с 1 изоцентром), ПД=54 Гр по 100% изодозной кривой.

При дальнейшем наблюдении пациентки в течение 6 мес после операции сохраняются эмоционально-личностные и когнитивные расстройства, гипопитуитарных нарушений нет, гипонатриемия 126–131 ммоль/л (рис. 2).

**Figure:**
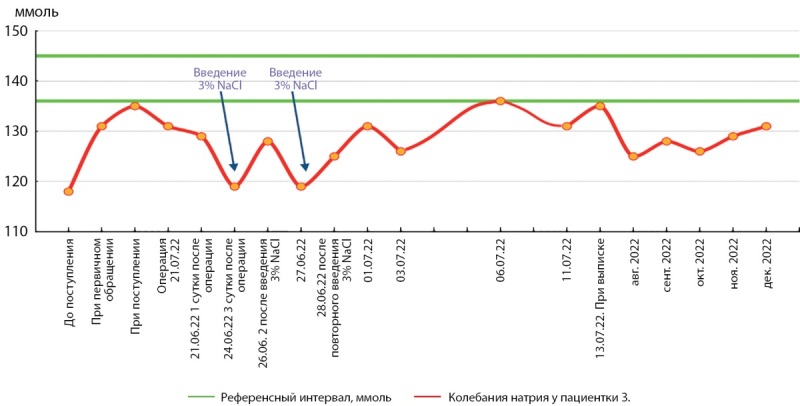
Рисунок 2. Динамика уровня натрия в крови у пациентки З.

## ОБСУЖДЕНИЕ

Гипонатриемия диагностируется при снижении уровня натрия менее 135 ммоль/л. Истинную гипонатриемию с низкой осмоляльностью плазмы следует отличать от псевдогипонатриемии, вызванной гиперлипидемией, гипергликемией или гиперазотемией при почечной недостаточности. В большинстве случаев умеренное снижение уровня натрия протекает бессимптомно. Клинические проявления обычно возникают при гипонатриемии менее 120–125 ммоль/л, в особенности если происходит ее быстрое развитие. Частой причиной снижения уровня натрия является синдром неадекватной секреции антидиуретического гормона (СНСАДГ), основным патогенетическим лечением которого является ограничение приема жидкости [1][2].

Первое сообщение о пациенте со стойкой гипонатриемией с отсутствием эффекта при ограничении потребления жидкости с сохранной функцией почек датируется 1976 г. Это состояние было названо синдромом “reset osmostat” — синдромом переустановки (перестройки/сброса) осмостата (СПО) и затем классифицировано как тип СНСАДГ [3].

При анализе литературы часто встречается разделение СНСАДГ на типы аномального высвобождения АДГ. Тип А — классический, когда наблюдается избыточная секреция АДГ с потерей зависимости от осмоляльности плазмы. Тип В — это редко встречающийся «сброс осмостата», что в настоящее время считается изменением нормального порога осмоляльности плазмы, вызывающим хроническую гипонатриемию. Секреция АДГ происходит при более низкой осмоляльности плазмы, чем обычно. Дальнейшее подавление АДГ возникает при осмоляльности плазмы ниже нижнего порога сброса и гипергидратации, что защищает от прогрессирования тяжелой гипонатриемии [4–7]. Тип С характеризуется неспособностью подавить секрецию АДГ при осмоляльности плазмы ниже осмотического порога, что происходит при дисфункции тормозных нейронов в гипоталамусе и приводит к стойкой базальной секреции АДГ, а тип D — нефрогенный СНСАДГ, редкая форма нарушения водного баланса с Х-сцепленным типом наследования с развитием мутаций гена рецептора АДГ 2-го типа (AVPR2), в результате которых нарушается реабсорбция свободной жидкости с развитием тяжелой симптоматической гипонатриемии [4][5][8][9].

Гипонатриемический сброс осмостата имеет низкий нормальный порог осмоляльности плазмы (обычно 280 мОсм/кг или менее), снижение уровня натрия менее 135 ммоль/л, что вызывает повышение АДГ при более низкой осмолярности плазмы с нормальным диурезом и неповрежденной способностью почек к концентрации мочи [1].

В литературе были описаны случаи СПО при передозировке препаратами десмопрессина, беременности, тяжелых инфекциях (например, ВИЧ-ассоциированных пневмониях, туберкулезе), квадриплегии, психозах, кровоизлиянии в мозг, энцефалите, алкоголизме, недоедании, злокачественных образованиях [3][4][10–13]. При этом клинических симптомов гипонатриемии чаще всего не наблюдалось, и обычно СПО диагностировался случайно и не требовал лечения. Однако успешное лечение некоторых обратимых состояний (например, туберкулеза или пневмонии) обычно приводит к нормализации осмостата [3].

Как известно, различают три механизма развития гипонатриемии: нарушение экскреции воды, чрезмерная потеря или недостаточное потребление натрия. Предположительным патогенетическим механизмом развития СПО является изменение метаболизма осморецепторов при длительном снижении уровня осмоляльности сыворотки, при котором происходит секреция АДГ, то есть они сохраняют нормальную реакцию на изменения осмоляльности сыворотки, хотя порог высвобождения АДГ снижен. Следовательно, уровень натрия в сыворотке крови ниже нормы, но стабилен, поскольку сохраняется способность выводить воду. [10]

При диагностике гипонатриемических состояний проводится исследование натрия, осмоляльности плазмы, а также сывороточных уровней других электролитов (калия, хлорида и бикарбоната), креатинина, мочевины, глюкозы, мочевой кислоты, общего белка и триглицеридов. Кроме того, обязательно определение уровней тиреотропного гормона, свободного тироксина и кортизола для исключения сопутствующей эндокринопатии. После подтверждения гипонатриемии и гипоосмолярности необходима оценка осмоляльности мочи, что позволит оценить способность почек концентрировать мочу. Естественная реакция почек на гипонатриемию — выработка максимально разбавленной мочи (осмоляльность мочи менее 100 мОсм/кг, удельный вес 1,003 или меньше). Если моча разбавлена, это указывает на то, что секреция АДГ полностью подавлена, что наблюдается у пациентов с первичной полидипсией, но также может быть характерна и для сброса осмостата. Гипонатриемия вряд ли разовьется в условиях интактного механизма разбавления мочи, однако в редких случаях все-таки встречается у пациентов с первичной полидипсией, которые употребляют большое количество воды (в отдельных случаях более 10–15 л/сут). В этих случаях тенденция к гипонатриемии будет выявлена и усилена сопутствующим нарушением выведения воды. Первичная полидипсия встречается у пациентов с дисфункцией центральной нервной системы или получающих антипсихотические средства, а также может быть связана с тошнотой или стресс- индуцированной секрецией АДГ. В других редких случаях гипонатриемия сочетается со сниженной осмоляльностью мочи, когда способность выводить воду снижается из-за неправильного питания. Это явление было описано у пациентов с хроническим алкоголизмом или при употреблении малого количества твердой пищи [2].

Важным диагностическим критерием гипонатриемии при CПО является отсутствие повышенной почечной экскреции натрия в моче.

Повышенная концентрация натрия в моче может возникать при некоторых заболеваниях почечной паренхимы, надпочечниковой недостаточности, СНСАДГ, соль-теряющем синдроме, метаболическом алкалозе, а также на фоне терапии тиазидными диуретиками (реже петлевыми) [1][2].

В нашей работе мы использовали биохимические показатели в крови и разовой порции мочи. У пациентки З. уровень натрия в разовой порции мочи был в пределах нормальных показателей (табл. 1).

Исследование фракционной экскреции мочевой кислоты (FE UA) как альтернативного маркера натрийуреза описано в отечественной работе Арутюнова Г.П. и соавт. и статье Feder J. и соавт. [3][14]. Она определяется по следующей формуле:

FE UA (%) = (U UA × P Cr) / (U Cr × P UA) × 100 %,

где UUA — уровень мочевой кислоты в моче, PCr — уровень креатинина плазмы, UCr — уровень креатинина в моче, PUA — уровень мочевой кислоты в плазме.

Применительно для данного клинического случая получен следующий результат:

FE UA (%) = (0,643 × 64) / (3 × 126) × 100 % = 10,2 % (табл. 1).

Информативным диагностическим маркером СПО является нормальная (4–11%) фракционная экскреция мочевой кислоты (FE UA). FE UA обычно повышена (>12%) у пациентов с гипонатриемией вследствие СНСАДГ и соль-теряющего синдрома и обычно снижена (<4%) при гиперволемической гипонатриемии: сердечной недостаточности, циррозе печени и нефротическом синдроме [14][15].

У пациентки З. по результатам расчетов подтверждено отсутствие повышенной почечной экскреции натрия.

При попытках коррекции гипонатриемии введение гипертонического раствора натрия хлорида при синдроме переустановки осмостата оказывает временный эффект на уровень натрия в крови. В моче после этого теста отмечаются преходящая гипернатриемия или нормонатриемия и гиперосмоляльность. В описанном нами случае назначение 3% гипертонического раствора пациентке приводило лишь к незначительному повышению или временной нормализации уровня натрия с последующим его снижением.

Наконец, окончательное подтверждение диагноза проводится после выполнения теста с водной нагрузкой для исключения классического синдрома СНСАДГ. Он состоит из пероральной водной нагрузки для подавления секреции АДГ из расчета 10–20 мл/кг массы тела, но не более 1,5 л, или данный объем жидкости вводят внутривенно в виде 0,9% раствора натрия хлорида. При выделении менее 65% выпитой жидкости через 4 ч после введения жидкости и/или выделение менее 80% выпитой жидкости через 5 ч после введения жидкости диагностируется СНСАДГ. При наличии синдрома сброса осмостата пациенты выделяют более 80% водной нагрузки в течение 5 часов, что отмечалось в нашем наблюдении [16].

Таким образом, можно выделить следующие диагностические критерии для диагностики СПО (табл. 2).

**Table:** Таблица 2. Диагностические критерии синдрома переустановки осмостата [3]

• Гипонатриемия. • Эуволемия. • Нормальная функция сердца, почек, надпочечников и щитовидной железы. • Сохраняющаяся гипонатриемия при введении гипертонического раствора натрия хлорида. • Cпособность почек выводить избыточную жидкость (положительный тест с водной нагрузкой). • Отсутствие повышенной почечной экскреции натрия. • Нормальная фракционная экскреция мочевой кислоты.

Прежде чем рассматривать терапию хронической гипонатриемии, следует устранить любую ее обратимую причину. Кроме того, у пациентов с синдромом сброса осмостата обычно наблюдается легкая или умеренная бессимптомная гипонатриемия, при которой происходит снижение порога как для высвобождения АДГ, так и возникновения жажды. Поскольку функция осморецепторов находится в норме в пределах нового порогового уровня, попытка повысить концентрацию натрия в сыворотке крови приведет к повышению уровня АДГ и вызовет у пациента чувство жажды, то есть реакцию, аналогичную той, которая наблюдается при ограничении жидкости у здоровых людей. Таким образом, попытка повысить концентрацию натрия в сыворотке крови может оказаться ненужной (учитывая отсутствие очевидных симптомов и риска более тяжелой гипонатриемии) и, вероятно, будет неэффективной [13].

## ЗАКЛЮЧЕНИЕ

Таким образом, у пациентки с хордоидной глиомой III желудочка была диагностирована хроническая гипонатриемия в результате развития СПО на основании отсутствия гипотиреоза и гипокортицизма, сохранной фильтрационной и концентрационной функции почек, а также сохраняющейся гипонатриемии при попытках коррекции уровня натрия гипертоническим раствором. Пробы с гипертоническим раствором и с водной нагрузкой позволили подтвердить СПО.

В данной ситуации умеренная гипонатриемия не расценивалась как жизнеугрожающая и, соответственно, не требовала коррекции. Необходимо помнить, что в первую очередь следует проводить лечение конкретного пациента, не стремясь к сведению показателей лабораторно-инструментальных данных в общепопуляционные рамки.

Дифференцированный подход к диагностике гипонатриемии различного генеза позволит определить наиболее оптимальную тактику ведения пациентов.

## УЧАСТИЕ АВТОРОВ. ДОПОЛНИТЕЛЬНАЯ ИНФОРМАЦИЯ

Источник финансирования. Работа выполнена по инициативе авторов без привлечения финансирования.

Конфликт интересов. Авторы декларируют отсутствие явных и потенциальных конфликтов интересов, связанных с публикацией настоящей статьи.

Участие авторов. Все авторы одобрили финальную версию статьи перед публикацией, выразили согласие нести ответственность за все аспекты работы, подразумевающую надлежащее изучение и решение вопросов, связанных с точностью или добросовестностью любой части работы

Согласие пациента. Пациент добровольно подписал информированное согласие на публикацию персональной медицинской информации в обезличенной форме.

## References

[cit1] Peng Goh K. Water and sodium balance management of hyponatremia. Am Fam Physician. 2004;69(10):2387-2394. 15168958

[cit2] (2008). The hyponatremic patient: A systematic approach to laboratory diagnosis. Nutrition in Clinical Practice.

[cit3] Musso CG, Feder J, Gomez JM, Serra-Aguirre F (2019). Reset osmostat: Facts and controversies. Indian Journal of Nephrology.

[cit4] Rigueto Larissa G., Santiago Henrique M., Hadad David J., Seguro Antonio Carlos, Girardi Adriana Castello C., Luchi Weverton M. (2022). The “new normal” osmotic threshold: Osmostat reset. Clinical Nephrology – Case Studies.

[cit5] Hannon M J, Thompson C J (2010). The syndrome of inappropriate antidiuretic hormone: prevalence, causes and consequences. European Journal of Endocrinology.

[cit6] Zerbe R, Stropes L, Robertson G (2003). Vasopressin Function in the Syndrome of Inappropriate Antidiuresis. Annual Review of Medicine.

[cit7] Robertson Gary L. (2008). Regulation of Arginine Vasopressin in the Syndrome of Inappropriate Antidiuresis. The American Journal of Medicine.

[cit8] Feldman Brian J., Rosenthal Stephen M., Vargas Gabriel A., Fenwick Raymond G., Huang Eric A., Matsuda-Abedini Mina, Lustig Robert H., Mathias Robert S., Portale Anthony A., Miller Walter L., Gitelman Stephen E. (2005). Nephrogenic Syndrome of Inappropriate Antidiuresis. New England Journal of Medicine.

[cit9] Makazan Nadezhda V., Zubkova Natalia A., Tiulpakov Anatolyi N. (2017). A case of nephrogenic syndrome of inappropriate antidiuresis caused by a mutation of the vasopressin type 2 receptor. Problems of Endocrinology.

[cit10] Vale Beatriz Maia, Morais Sofia, Mesquita Joana, Mimoso Gabriela (2015). Reset osmostat: a rare cause of hyponatraemia. BMJ Case Reports.

[cit11] Kahn Thomas (2011). Reset Osmostat and Salt and Water Retention in the Course of Severe Hyponatremia. Medicine.

[cit12] Leggott J, Almond D. Reset osmostat in a 47-year-old woman with cerebral palsy. J Am Board Fam Pract. 2001;14(4):317-319. 11458975

[cit13] Sterns RH. Treatment of hyponatremia: Syndrome of Inappropriate Antidiuretic Hormone Secretion (SIADH) and reset osmostat. UpToDate. 2022.

[cit14] Arutyunov G.P., Dragunov D.O., Sokolova A.V., Arutyunov A.G. Fraktsionnaya ekskretsiya mochevoĭ kisloty kak al'ternativnyĭ marker nizkogo urovnya natriĭureza // Klinicheskaya nefrologiya. — 2014. — №5. — S. 20-24.

[cit15] Pathophysiology. In: Alpern RJ, Hebert SC, editors. Seldin and Giebisch’s The Kidney (Fourth Edition). 2008. Vol. 2. P. 2113-2141.

[cit16] Pigarova E.A. Pervichnye i vtorichnye sindromy gipo- i gipernatriemii v endokrinologii, ikh sovremennaya diagnostika i lechenie: Dis. … doktor med. nauk. — M.: FGBU Natsional'nyi meditsinskii issledovatel'skii tsentr endokrinologii MZ RF; 2019.

